# Alleviating Surgeons’ Stress through Listening to Natural Sounds in a Half-Encapsulated Rest Space after an Operation: A Pilot, Longitudinal Field Study

**DOI:** 10.3390/ijerph191912736

**Published:** 2022-10-05

**Authors:** Yasushi Suko, Tomoharu Shindo, Kaoru Saito, Norimasa Takayama, Shin’ichi Warisawa, Tetsuya Sakuma, Masaaki Ito, Pasi Kytölä, Tapio Nummi, Kalevi Korpela

**Affiliations:** 1 Faculty of Social Sciences/Psychology, Tampere University, FI-33014 Tampere University, Finland; 2 Department of Natural Environmental Studies, Graduate School of Frontier Sciences, The University of Tokyo, 5-1-5 Kashiwanoha, Kashiwa-shi 277-8563, Chiba, Japan; 3 Department of Human and Engineered Environmental Studies, Graduate School of Frontier Sciences, The University of Tokyo, 5-1-5 Kashiwanoha, Kashiwa-shi 277-8563, Chiba, Japan; 4 Department of Landscape Architecture Science, Tokyo University of Agriculture, Sakuragaoka 1-1-1, Setagaya-ku, Tokyo 156-8502, Japan; 5 Department of Forest Management, Forestry and Forest Products Research Institute, 1 Matsunosato, Tsukuba 305-8687, Ibaraki, Japan; 6 Department of Architecture, Graduate School of Engineering, The University of Tokyo, 7-3-1 Hongo, Bunkyo-ku, Tokyo 113-8656, Japan; 7 Department of Colorectal Surgery, National Cancer Center Hospital East, 6-5-1 Kashiwanoha, Kashiwa-shi 277-8577, Chiba, Japan; 8 Faculty of Information Technology and Communication Sciences/Statistics, Tampere University, FI-33014 Tampere University, Finland

**Keywords:** restorative effect, natural sounds, surgeon, skin conductance level (SCL), Restoration Outcome Scale (ROS), Profile of Mood States (POMS), mental health, well-being

## Abstract

Background: Natural sounds are reportedly restorative, but most research has used one-off experiments conducted in artificial conditions. Research based on field experiments is still in its infancy. This study aimed to generate hypotheses on the restorative effects of listening to natural sounds on surgeons, representing professionals working in stressful conditions. Methods: Each of four surgeons (two experts and two residents) participated six times in an experiment where they took a 10-min break listening to natural sounds (four times) or without natural sounds (twice) after a surgical operation. We measured their skin conductance level, an indicator of sympathetic arousal, continuously during the break (measurement occasions *N* = 2520) and assessed their mood using two questionnaires before and after the break (*N* = 69 and *N* = 42). We also interviewed them after the break. Results: Based on statistical Linear Mixed-Effects modeling, we developed two hypotheses for further, more detailed studies: (H1) Listening to natural sounds after an operation improves surgeons’ mood. (H2) Inexperienced surgeons’ tension persists so long that the effect of natural sounds on their sympathetic arousal is negligible. Conclusions: This risk-free, easy-to-use means of stress alleviation through natural sounds could benefit highly-stressed people working indoors.

## 1. Introduction

Contact with nature is supposed to have a restorative effect [1,2]. The restorative effect refers to the effect that promotes restoration, or “the process of renewing, recovering, or reestablishing physical, psychological, and social resources or capabilities diminished in ongoing efforts to meet adaptive demands” [3]. This effect could also appear even if people do not actually visit the natural environment [4].

As one aspect of restoration, many studies have described the restorative effect of natural sounds (e.g., birdsong, sound of a running river, sound of a fountain) on stress recovery. After psychological stress, physiological recovery of sympathetic activation may be “faster during exposure to pleasant nature sounds than to less pleasant noise” [5]. Stress recovery can be “facilitated by the addition of sounds of nature to a virtual green environment in a laboratory setting” [6]. Certain bird sounds may “aid perceived restoration from stress or fatigue by encouraging positive affect and reduced arousal, alternative and effortless attentional focus to novel stimuli, and connection to nature” [7]. 

The benefit of natural sounds has also been discussed in the context of sound therapy. One study focused on nature-based rehabilitation and suggested that “the presence of natural sounds along with sounds from walking materials, social quietness, and absence of technological sounds could be regarded as important prerequisites for mental restoration and recovery from stress, and therefore considered essential aspects for designing restorative places” [8]. Another study found that listening to natural sounds for 90 min significantly decreased systolic blood pressure, diastolic blood pressure, anxiety, and agitation levels of patients under mechanical ventilation support [9].

Two theories highlight the need to empirically assess the restorative effect of natural sounds on people’s mood and physiology: The Attention Restoration Theory (ART) and the Stress-Reduction Theory (SRT). The ART explains the mechanisms of the restorative effect of contact with nature based on the concept of *directed attention* [10]. The SRT complements the theoretical perspective of the ART. The SRT suggests that a positive initial affective and physiological response, which may often derive from unthreatening natural stimuli, should reduce stress-related feelings and taxing physiological mobilization [11,12]. 

Listening to natural sounds that meet both the ART’s and SRT’s prerequisites for being restorative could facilitate mitigating people’s occupational stress and ameliorating their mood states. Such sounds would be especially beneficial to those whose professions require maintaining directed attention and activating the sympathetic nervous system for a long time. Healthcare professionals such as surgeons would be a good example because they are continuously subject to occupational stress due to long working hours and massive responsibility for patients. A substantial number of surgeons are struggling with personal and professional distress at a level that should be of concern [13]. Therefore, this study focuses on increasing the coping opportunities to alleviate surgeons’ stress in such circumstances using natural sounds.

As most findings on the mental benefits of natural sounds derive from laboratory experiments, we designed a field experiment to extend such findings to the actual stressful work environment. The experiment was conducted during surgeons’ working hours without impeding their work. First, we designed an experiment where participants repeatedly listened to several natural sound clips. The majority of the existing studies on the benefits of natural sounds is based on one-off experiments [5,14,15,16], but these simplified experimental designs do not suffice to investigate the complex mechanisms of people’s coping and restoration that restorative natural sounds provide. For example, in a setting where participants are supposed to listen repeatedly to natural sounds, it would be better to provide several sound clips and allow the participants to change clips freely during the intervention to avoid boredom. 

Second, we considered surgeons’ experience levels because these may affect their coping strategies after a surgical operation, as is the case with athletes before or after their performance. A recent review article on sports suggests that elite athletes who exhibit low levels of the negative mood states, such as anxiety, anger, depression, confusion, and fatigue, and increased levels of vigor will evidence greater athletic success than those who do not [17]. Therefore, if surgeons have a similar tendency, inexperienced surgeons may be tense during an operation, and their acute stress may persist longer than experienced surgeons’ acute stress. For example, after an operation, experienced surgeons may recover from acute stress and be ready for their next task by listening to natural sounds during a short break, while inexperienced surgeons may not. Such a difference could affect the level of their restorative experience when listening to natural sounds. Thus, we purposefully designed a study that targeted surgeons at different levels (e.g., experts and residents, about which are described in Section 2 in more detail).

### Objectives

To overcome the research gaps mentioned above, we designed a pilot, longitudinal study intended to generate hypotheses on the restorative effect of natural sounds on surgeons, who represent professionals working in a stressful environment. The term “longitudinal” in this study refers to its use as in, e.g., Applied Longitudinal Analysis [18]; it suggests longer measurement intervals (e.g., days) rather than a relatively short amount of time (e.g., minutes, hours). The applied longitudinal analysis is described in detail under Section 2.

Our research interest lies in (1) whether listening to natural sounds after a surgical operation reduces surgeons’ physiological and psychological stress and (2) whether surgeons’ stress in this situation proceeds in a different way depending on their level of experience. We aimed to examine all cases in a many-sided way combining physiological and self-report data based on a longitudinal design. We also categorized surgeons into two groups according to their levels of experience, but its primary reason was to observe in-depth how surgeons’ physiological responses and self-report data change over time *within* each of these groups. Therefore, the between-groups comparison (i.e., more experienced surgeons vs. less experienced surgeons) was not the primary reason for creating this experimental design.

## 2. Materials and Methods

### 2.1. Acoustic Stimuli

Five types of 10-min long natural sounds were used: (1) loud birdsong, (2) gentle birdsong, (3) the sound of a running river, (4) the sound of wind, and (5) the sound of rain. They were extracted from the Cyberforest Links [19]. This database is based on the Cyberforest Project, which has been live streaming and recording sounds in several forests in Japan since 1995. Participants were able to choose natural sounds and adjust their volume freely during a 10-min break after an operation. Regarding the rationale for the length of the break, see Supplementary Materials.

We decided to use these five natural sounds for the following reasons. First, we intended to use sound clips of *geophony*, or “non-biological sounds that occur in natural habitats,” and *biophony*, or “the special collective voices of the natural world” [20]. In our current study, the sounds of a running river, wind, and rain represent geophony and birdsongs represent biophony. In addition, as described in Section 1, birdsong has been claimed to be restorative. Therefore, we included two different clips of birdsongs, thereby comparing the effects/preferences of loud and gentle birdsong.

### 2.2. Sound Playback System

In this study, participants listened to natural sounds (in the experimental condition) or spent time without listening (in the control condition) sitting inside the Sound Cocoon, a half-encapsulated sound playback apparatus developed by Pioneer Corporation. The Sound Cocoon enabled surgeons to have a break more comfortably by partially insulating ambient noise (e.g., the sound of a TV or others’ conversation in the rest space) in both conditions (experimental/control). At the same time, its front part was kept open, which allowed other surgeons and nurses, in case of emergency, could contact those who were resting. During the break in the experimental condition, the participants were allowed to change the volume of natural sounds freely using an iPad (Apple Inc., Los Altos, CA, USA) attached to the Sound Cocoon, while the default sound type was randomized.

### 2.3. Physiological Measurement: Skin Conductance Level (SCL)

The skin conductance level (SCL) refers to the “tonic level of electrical conductivity of skin” and this is one of the measures of electrodermal activity (EDA), a physiological indicator [21]. The EDA is claimed to have advantages over many other psychophysiological measures, especially in terms of being “relatively inexpensive to record” and “completely harmless and risk-free” [21], and has been widely used. The SCL is considered one of “the most useful electrodermal measures in the context of continuous stimuli” because it “can be measured on an ongoing basis over relatively long periods of time” [21]. Given that we aimed to assess the continuous change in surgeons’ physiological responses during a 10-min break and that we should not risk causing harm to them by measurements in the clinical setting, we decided to use the SCL in this study. 

In studies on the physiological and psychological effects of natural sounds on people (e.g., [14,16,22]), the SCL corresponds to the extent to which these acoustic stimuli affect the listeners’ physiological response, namely, the degree of sympathetic nervous activity. The higher a participant’s SCL, the higher his/her level of arousal, and vice versa. 

To the best of our knowledge, this study is the first to describe the change in surgeons’ SCLs during a break immediately after a real surgical operation. Although a recent systematic review published in 2017 [23] showed that five studies had been conducted using skin conductance to investigate surgeons’ and surgical trainees’ acute stress, all five studies were based on surgical simulation. In addition, as these studies focused mainly on measuring how surgeons’ stress changed during an operation compared to a resting condition before an operation (e.g., [24]), research on the effects of a break after an operation on surgeons’ SCLs is lacking.

### 2.4. Questionnaire 1. Restoration Outcome Scale Japanese Edition (ROS-J)

The Restoration Outcome Scale (ROS) provides a measure of general restorative experiences based on six items. Three of the items reflected relaxation and calmness, one item reflected attention restoration, and two items reflected clearing one’s thoughts [25]. ROS measured before a field experiment can refer to the baseline, i.e., a person’s potential state (stressed or relaxed) [26]. Fujisawa and Takayama developed a ROS Japanese version (ROS-J) and confirmed its validity and reliability, and revealed that the virtual forest environment could reduce people’s stress [27]. In this experiment, we used the ROS-J three times: (1) before an operation, (2) after an operation (before a break), and (3) after a break, and considered the score at (2) as reference (baseline).

### 2.5. Questionnaire 2. Profile of Mood States 2-A Short (POMS2-A Short)

The Profile of Mood States Second Edition (POMS2) comprises self-report scales that allow for the quick assessment of transient, fluctuating feelings and enduring affect states [28]. The Japanese versions of the POMS2 were also developed [29]. A short form of POMS2 Adult (POMS2-A Short), which is for those who are over the age of 18, consists of 35 items with a five-point Likert scale ranging from 0 (not at all) to 4 (extremely). In this study, we used the Japanese version of the POMS2-A Short. We conducted this questionnaire survey twice in each experiment: (1) after an operation and (2) after the break because it takes three to five minutes to answer the POMS2-A Short. Answering it before the operation would be a burden to surgeons.

### 2.6. Interview Survey

An interview survey was conducted at the end of each experiment. Eight main questions, which are described below, and additional questions were occasionally asked or some of the main questions were omitted depending on the surgeons’ answers or busyness. This survey aimed to investigate the surgeons’ detailed impressions and subjective evaluations of the sounds, which could not be assessed by questionnaires. The questions are as follows:

- Q1. Were you nervous during the operation today?
- Q2. Please rate how nervous you were using a 1–10 Likert Scale (1 = not nervous at all to 10 = very nervous)
- Q3. Please rate the difficulty level of today’s operation that you expected before it began by a 1–10 Likert Scale (1 = easy to 10 = difficult)
- Q4. Please rate the difficulty level of today’s operation that you felt after it finished by a 1–10 Likert Scale (1 = easy to 10 = difficult)
- Q5. Please describe your preference for the type of natural sounds and the volume (this question was asked only in the experimental condition, in which the surgeons listened to natural sounds)
- Q6. Which do you prefer, having natural sounds or not having them during the break?
- Q7. Which do you prefer, resting inside or outside of the Sound Cocoon? Also, would your answer to this question differ depending on whether or not there were natural sounds?
- Q8. How was the duration of the 10-min break? Was it “long,” “moderate,” or “short”?

### 2.7. Sample and Experimental Protocol

The study was conducted at a hospital from October 2019 to February 2020. Four surgeons consisting of two experts and two residents (33–45 years old) participated in the experiment. Experts can perform surgical operations at their own discretion, while residents need experts’ supervision to do it. We informed all the participants of the objectives and protocols of the experiment and obtained their informed consent before the experiment. This study was reviewed and approved by the Ethics Review Committee of the University of Tokyo (the approval number for the original application is 18-206, and that for an amendment application is 21-173). All of them were male, and they took part in the experiment six times, four times in the experimental condition (with natural sounds) and twice in the control condition (without natural sounds). Therefore, all the surgeons experienced both conditions (experimental/control).

It should be emphasized that, in this study, statistical analysis is not based on the number of participants per se (*N* = 4); the analysis is based on the number of observations in the repeated measures experimental design, i.e., *N* = 2520 for the physiological measurement, *N* = 69 and *N* = 42 for two types of questionnaires’ scores. The details of the number of observations will be explained in Section 2.9, “Sample Size Justification”.

We excluded the data obtained from one experiment in the experimental condition for Surgeon R1 because, on that day, the operation involved a closure of a colostomy. This type of surgery lasts only 15–30 min and is technically much easier than usual operations, which last several hours. We concluded that this exceptional operation did not work as a stressor. Therefore, the total number of valid trials was 23, comprising 15 trials in the experimental condition and eight trials in the control condition. We conducted one experiment for one surgeon on a day. Table 1 shows the details.

**Figure 1:**
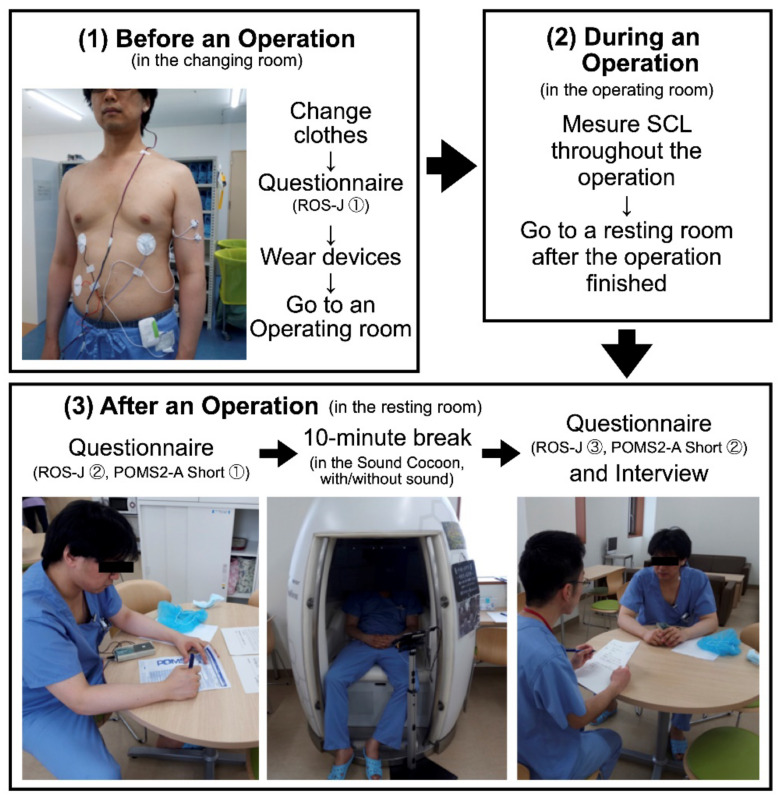
Experimental protocol of this study. Note. The Sound Cocoon is in the bottom center picture. Although the ECG data was used for another study, we measured the surgeons’ SCLs and ECGs from *before an operation* until *after a break* because this was the only possible way. Surgeons did not have enough time to attach or take off electrodes somewhere between *after an operation* and *after a break* because, after an operation, they were usually supposed to visit the intensive care unit (ICU), where the postoperative patient was sent, to observe her or his condition. Exceptionally, during the period of our experiment, the surgeons came to the resting room and engaged in a 10-min intervention just before going to the ICU (note: This protocol did not put the patient at risk because more than one surgeon took charge of each operation and only one of them participated in our experiment at a time. The other surgeons could visit the ICU immediately after an operation ended). This being so, we decided to put all the electrodes on their body *before an operation* and take them off *after a break*.

Figure 1 describes the experimental protocol of this study. After a morning meeting, a surgeon went to a changing room and changed his clothes. After that, he answered the first ROS-J questionnaire, and then put sensors for physiological measurement on. Two electrodes for the SCL measurement were placed on the left upper arm, one of which was 15 cm below the left shoulder and the other was 5 cm below the first one. We began to make a measurement of the SCL soon after the electrodes were attached to the surgeon’s body. After finishing putting on the electrodes, he went to an operating room and a surgical procedure began. 

After the procedure finished, the surgeon went to a resting room for surgeons and nurses, where the Sound Cocoon was set up, and answered the second ROS-J questionnaire and the first POMS2-A Short. Soon after that, he took a break sitting inside the Sound Cocoon for 10 min. In the experimental condition, he listened to natural sounds during the break. He was able to select natural sounds from the five alternatives and was able to change the volume freely. In contrast, in the control condition, he sat still in the Sound Cocoon without any acoustic stimuli, but he still could hear ambient sounds (e.g., nurses’ conversational voices or the sound of a TV in the same resting room). Regarding the counterbalancing of the experiencing order (experimental/control), see Supplementary Materials. After the break, the surgeon went out of the Sound Cocoon and answered the third ROS-J and the second POMS2-A Short. After answering these questionnaires, we conducted an interview survey to investigate the surgeon’s subjective impressions of the experiment, which were difficult to evaluate using physiological measurements or psychological questionnaires. After the interview survey, the electrodes for physiological measurement were removed.

As the picture on the upper left in Figure 1 shows, we also measured surgeons’ physiological responses during an operation focusing on the SCL and electrocardiogram (ECG). However, those results will be reported in another article whose interest lies in how the surgeons’ physiological stress changes in each operation phase.

### 2.8. Statistical Analyses

The changes in the SCL, ROS-J, and POMS2-A Short over time were analyzed using Linear Mixed Effects models (LME models) [30]. Model effects are divided into fixed and random effects, assuming that random effects and random errors are independent and follow the normal distribution. Modeling in this context involves selecting the fixed and random parts of the model and choosing covariance structures related to random effects and random errors. The Akaike Information Criterion (AIC) was used in random part model selection. The LME models were applied to examine the change in the SCL, ROS-J, and POMS2-A Short over time. The ROS-J (sub)scales scores, including the total score, were treated as approximations of continuous variables because they are based on symmetric seven-point Likert scale variables. Likewise, the POMS2-A Short (sub)scales scores, including TMD, were treated as approximations of continuous variables because they are based on symmetric five-point Likert scale variables. In the analytical phase, we converted these POMS2-A Short (sub)scales scores to T scores, the standardized scale [31,32]. The marginal significance of the fixed effect coefficients of interest and the 95% confidence intervals were based on the restricted maximum likelihood estimator large sample theory.

For the SCL, time was considered as a continuous variable during the 10-min break. For ROS-J, the measurement occasion (MO) was considered a three-level categorical variable referring to the timing when the surgeons completed the questionnaire: *MO1* = before an operation, *MO2* = after an operation (before a break), and *MO3* = after a break. For POMS2-A, MO was a two-level categorical variable (i.e., *MO2* and *MO3*) because this questionnaire was not filled out before an operation. Random effects in these models account for the surgeon-level dependencies in the repeated measures. Random effects were assumed to be independent and have the same variance.

Statistical software used was lme function from R’s nlme package [33], version 3.1.149. The data set used for the analysis is shown on an online repository [34].

### 2.9. Sample Size Justification

Existing studies on acute stress in surgeons during surgical operations have relied on a small number of participants, notably under five (*N* < 5) [23]. One of the primary reasons for this was to avoid inter-individual (physiological) variations [35]. It is also challenging for surgeons to participate in a scientific study that interrupts their everyday routines. For these reasons, our study included four surgeons, thereby minimizing interference with surgeons’ daily work. However, as this study was longitudinal, the number of measurements on which our statistical analysis was based was much larger than the so-called sample size per se, which usually refers to the number of participants (for example, see [35,36]).

In this study, we measured four surgeons’ SCLs and mood states multiple times in each trial and converted these data to the long format for the LME model analysis. The unit of analysis is the measurement occasion (MO), not the trial (day). The MO refers to the timing of the measurement (when a measurement was made) in each trial; three MOs for ROS-J (before an operation, after an operation, and after a break), two for POMS2-A Short (after an operation and after a break), and 120 for the SCL (each 5 s during a 10-min break). Therefore, the total numbers of MOs throughout the entire trials were as follows: 69 for ROS-J (3 MOs per trial × 23 trials); 42 for POMS2-A Short (2 MOs per trial × 21 trials); 2520 for the SCL (120 MOs per trial × 21 trials). Here, POMS2-A Short and the SCL measurements were unsuccessful in two out of the 23 trials, as described in Table 1. 

Post hoc power calculations were conducted using Pwr function from R’s nlmeU package, version 0.70.3 [37]. It revealed that, based on effect sizes observed in the study, none of the statistical tests for the questionnaire scores provided the recommended 0.80 statistical power level; at 0.05 level they ranged from 0.55 to 0.78. In contrast, the post hoc powers for all the SCL tests were higher than 0.80 (=1.0).

## 3. Results

### 3.1. Acoustic Stimuli

**Figure 2:**
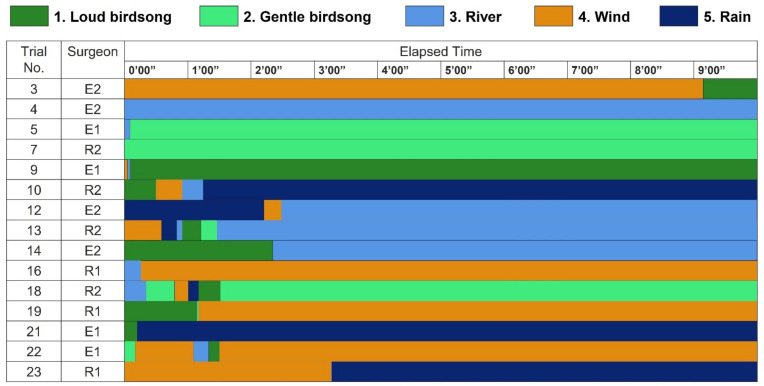
Surgeons’ patterns of listening to natural sounds.

Figure 2 shows the surgeons’ patterns of listening to natural sounds during a break. All the surgeons finished adjusting the type of natural sounds during the first 3 min 30 s, whereas they did not make any change during the latter half of the 10-min break, except for Surgeon E2 in Trial No. 3. Therefore, we defined “a listened sound” as the one he listened to from 5 to 6 min after the break started each day. The sounds that were most frequently listened to were the sound of a running river and wind, followed by gentle birdsong and the sound of rain, and then loud birdsong. That is, constant natural sounds tended to be preferred over intermittent sounds. We will discuss this difference in Section 4, considering surgeons’ answers in the interview.

**Figure 3:**
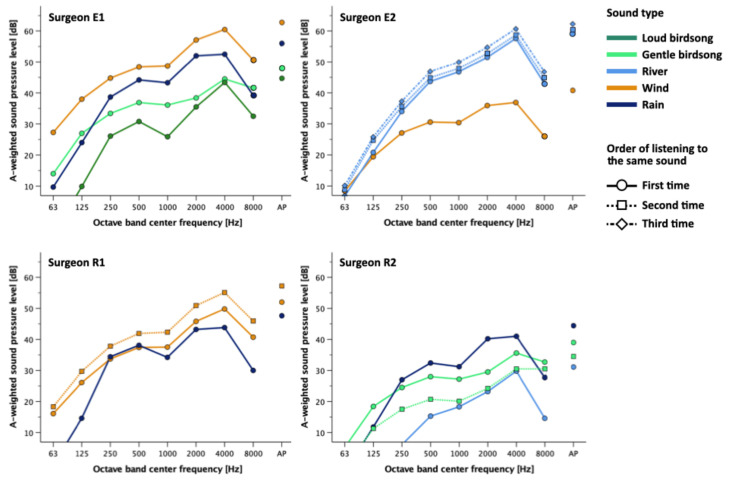
Type of sound and the sound frequency characteristics of equivalent continuous A-weighted sound pressure levels in each experiment. Note. AP = All Pass.

Figure 3 shows the A-weighted sound pressure level at each octave-band center frequency for the one-minute extract (5′00″-6′00″) of each 10-min sound clip for each surgeon. We determined the sound pressure level by measuring the transfer characteristics of the audio system in advance and the line level of the audio signal during playback. Surgeon E1 listened to loud birdsong at 45 dB and gentle birdsong at 48 dB, which were relatively calm, whereas he listened to wind at 63 dB and rain at 56 dB. Surgeon E2 listened to wind at 41 dB, and river at 59, 60, and 62 dB. Surgeon R1 listened to wind at 52 and 57 dB, and rain at 48 dB. Surgeon R2 listened to gentle birdsong at 35 and 39 dB, river at 31 dB, and rain at 44 dB. All types of natural sounds had dominant components in the 4 kHz band, which shows that the high-frequency range is prominent. Regarding the trend of the sound pressure levels by type of sounds, all the surgeons listened to low levels of the birdsongs. As for the interparticipant difference, Surgeon R2 listened to natural sounds at lower levels compared to the other surgeons. 

### 3.2. Skin Conductance Level (SCL)

In the LME model, a two-level categorical variable *SC* was used to differentiate the expert surgeons (E1 and E2) from the resident surgeons (R1 and R2). Likewise, a two-level categorical variable *NS* was used to differentiate the break with natural sounds from the break without natural sounds. The unit of the time variable *TIME* was five seconds. Here, *TIME* is the measurement occasion for the SCL. The estimation for the SCL is expressed in Equation (1):

Equation (1). LME Model for the SCL

(1) $$SCL_{ijk}=\beta_{0}+\beta_{1}\times NS_{i}+\beta_{2}\times SC_{i}+\beta_{3}\times TIME_{i}+\beta_{4}\times NS_{i}\times TIME_{i}+\;\beta_{5}\times SC_{i}\times TIME_{i}+\beta_{6}\times NS_{i}\times SC_{i}\times TIME_{i}+b_{1i}+b_{2ij}+\epsilon_{ijk}$$

Here, *i* refers to a surgeon (E1, E2, R1, or R2). This equation has two random effects; $b_{1i}$ is the grouping at the surgeons’ level (expert/resident), and $b_{2ij}$ is the grouping at nested surgeons’ working day *j* level.

The parameter estimates in this model (see Table 2) express the increase of the SCL, but our interest lies in the reduction of the surgeons’ SCLs (i.e., reduction of their sympathetic nervous system activity). Therefore, the number signs of the parameter estimates shown in Table 2 are reversed in the following three paragraphs.

The best estimate of the reduction of the expert surgeons’ SCLs in each five-second unit (i.e., *TIME*) during the break with natural sounds was 0.025 − 0.021 − 0.022 + 0.034 = 0.016 μS. For a 10-min break, this means a 120 × 0.016 μS = 1.92 μS reduction in the SCL. Likewise, in the control condition (i.e., without natural sounds), the reduction of the experts’ SCLs after a 10-min break was 120 × (0.025 − 0.022) = 0.23 μS.

The best estimate of the reduction of the resident surgeons’ SCLs by *TIME* during the break with natural sounds was 0.025 − 0.021 = 0.004 μS. For a 10-min break, this means a 120 × 0.004 μS = 0.48 μS reduction in the SCL. In the control condition, the reduction in the residents’ SCLs after a 10-min break was 120 × 0.025 = 3.0 μS.

**Figure 4:**
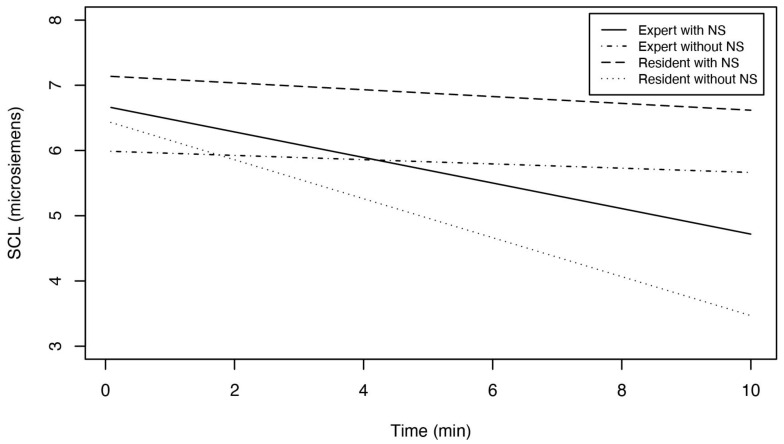
Prediction of the surgeons’ SCLs during the 10-min break.

To sum up, compared to the control condition, listening to natural sounds for 10 min enhanced the reduction of the experts’ SCLs by 1.92 − 0.23 = 1.69 μS. In contrast, natural sounds inhibited the reduction of the residents’ SCLs by 3.0 − 0.48 = 2.52 μS. Figure 4 illustrates these results as the prediction of the SCL for the surgeons during the 10-min break. In Section 4, we will further review the difference between the experts’ and residents’ SCLs, including whether or not these changes in the SCL are clinically significant.

### 3.3. ROS-J

We compared the difference in the surgeons’ ROS-J total score, including subscales, between *after an operation* and *after a break* in each condition (with/without natural sounds). We employed two LME models; Model 1 was to compare the scores between the two conditions (with natural sounds/silence) in general without differentiating the experts from the residents, whereas Model 2 was to do it in each surgeon class (expert/resident) separately. The estimations for ROS-J in Models 1 and 2 are expressed in Equations (2) and (3):

Equation (2). LME Model 1 for ROS-J

(2) $$ROS-J_{ijk}=\beta_{0}+\beta_{1}\times MO1_{i}+\beta_{2}\times MO3_{i}+\beta_{3}\times NS_{i}+\beta_{5}\times MO1_{i}\times NS_{i}\;+\beta_{6}\times MO3_{i}\times NS_{i}+b_{1ij}+\epsilon_{ijk}$$

Equation (3). LME Model 2 for ROS-J

(3) $$\begin{array}{cc} ROS-J_{ijk} & =\beta_{0}+\beta_{1}\times MO1_{i}+\beta_{2}\times MO3_{i}+\beta_{3}\times NS_{i}+\beta_{4}\times SC_{i}\\ & +\beta_{5}\times MO1_{i}\times NS_{i}+\beta_{6}\times MO3_{i}\times NS_{i}+\beta_{7}\times MO1_{i}\times SC_{i}\\ & +\beta_{8}\times MO3_{i}\times SC_{i}+\beta_{9}\times NS_{i}\times SC_{i}\\ & +\beta_{10}\times MO1_{i}\times NS_{i}\times SC_{i}+\beta_{11}\times MO3_{i}\times NS_{i}\times SC_{i}+b_{1ij}\\ & +\epsilon_{ijk} \end{array}$$

Note: *MO* = measurement occasion, *NS* = with or without natural sounds, *SC* = expert surgeons or resident surgeons. In these models, we chose *MO2* (*after an operation*) as the reference level. Indices *i*, *j*, and *k* refer respectively to a surgeon, a measurement occasion, and repetition in a particular surgeon and a measurement occasion.

In Model 1, in general, a break with natural sounds significantly increased the surgeons’ ROS-J total scores by 6.96, 95% CI [1.94, 11.98] (see $\beta_{6}$ in Table 3). Here, the increase in these scores suggests improvements in mood states. In contrast, a break without natural sounds did not significantly change any ROS-J scores (see $\beta_{2}$ in Table 3).

In Model 2, compared to the residents at *MO2* (*after an operation*), the experts’ ROS-J total scores did not change significantly after a break, regardless of with or without natural sounds (see $\beta_{8}$ and $\beta_{11}$ in Table 3). A break with natural sounds increased the residents’ ROS-J total scores by 7.86, 95% CI [−0.05, 15.8], with *p* = 0.051 (see $\beta_{6}$ in Table 3). Although this change was not significant with the significance level α=0.05, the *p*-value was close to 0.05, and this result is of consideration. Regarding the results of each sub-scale (Q1–Q6), see Tables S1 and S2 in Supplementary Materials.

### 3.4. POMS2-A Short

As in the case of ROS-J, we compared the difference in the surgeons’ POMS2-A Short Total Mood Disturbance (TMD) scores, including subscales, between *after an operation* and *after a break* in each condition (with/without natural sounds) based on LME models, setting *after an operation* as the reference level. Here, *before an operation* is not included because the surgeons did not fill out POMS2-A Short before an operation, as was explained in Section 2. We used the same models that were for ROS-J; Model 1 was to compare the scores between the two conditions (with natural sounds/silence) in general without differentiating the experts from the residents, whereas Model 2 was to do it in each surgeon class (experts/residents) separately. The estimations for POMS2-A Short in Models 1 and 2 are expressed in Equations (4) and (5):

Equation (4). LME Model 1 for POMS2-A Short

(4) $$POMS2A\;Short_{ijk}=\beta_{0}+\beta_{1}\times MO3_{i}+\beta_{2}\times NS_{i}+\beta_{4}\times MO3_{i}\times NS_{i}+b_{1ij}+\epsilon_{ijk}$$

Equation (5). LME Model 2 for POMS2-A Short

(5) $$\begin{array}{c} POMS2A\;Short_{ijk}=\beta_{0}+\beta_{1}\times MO3_{i}+\beta_{2}\times NS_{i}+\beta_{3}\times SC_{i}+\beta_{4}\times MO3_{i}\times NS_{i}\\ +\beta_{5}\times MO3_{i}\times SC_{i}+\beta_{6}\times NS_{i}\times SC_{i}+\beta_{7}\times MO3_{i}\times NS_{i}\times SC_{i}+b_{1ij}+\epsilon_{ijk}\; \end{array}$$

Note: *MO* = measurement occasion, *NS* = with or without natural sounds, *SC* = expert surgeons or resident surgeons. In these models, we chose *MO2* (*after an operation*) as the reference level. Indices *i*, *j*, and *k* refer respectively to a surgeon, a measurement occasion, and repetition in a particular surgeon and a measurement occasion.

In Model 1, in general, a break with natural sounds significantly increased the surgeons’ POMS2-A Short TMD scores by −5.24, 95% CI [−10.15, −0.33] (see $\beta_{4}$ in Table 3). Here, the decrease in the TMD scores suggests improvements in mood states. In contrast, a break without natural sounds did not significantly change TMD scores (see $\beta_{1}$ in Table 3). 

In Model 2, the residents’ TMD scores significantly decreased by 7.22 on average, 95% CI [−13.99, −0.44], after a break with natural sounds (see $\beta_{4}$ in Table 3), but not after a break without natural sounds (see $\beta_{1}$ in Table 3). Compared to the residents *after an operation*, the experts’ TMD scores *after a break* was not significantly different, regardless of with or without natural sounds. Regarding the results of each sub-scale, see Tables S3 and S4 in Supplementary Materials.

### 3.5. Interview

Regarding Q6, “Which do you prefer, having natural sounds or not having them during the break?” Surgeons E1 and R1 answered “natural sounds” from the beginning of the experiment, whereas Surgeons E2 and R2’s answers changed as time passed. The latter eventually came to prefer a break with natural sounds. Surgeons E1 and R1 also described their preferences for natural sounds. Surgeon E1 preferred different types of natural sounds depending on his mood states; he usually liked birdsong (Trial No. 5), but he liked the sound of rain and disliked birdsong when he was in a bad mood (Trial No. 21). On the day of Trial No. 21, he said he chose the sound of rain because he wanted to be beaten by rain to get rid of his bad mood. Surgeon R1 preferred the sound of the wind (Trial No. 16 and 19) or the sound of rain (Trial No. 23). He preferred the sound of wind because of its constancy. In contrast, he felt birdsong was intrusive and disliked it (Trial No. 19 and 23). For more details, see Tables S5–S8 in Supplementary Materials.

## 4. Discussion

Before entering into the discussion on the results for the SCL, it should be noted that there is no stand-alone criterion for the *clinical* significance of the change in the SCL due to “large variability because of extraneous individual differences” [21]. This fact necessitates comparing the SCL with other measurements, such as other physiological indicators or the responses in self-report questionnaires. For example, the above-mentioned study on surgeons’ SCLs [24] tested the statistical significance of the change in the SCL and discussed its clinical meaning in parallel with subjective ratings. Another example is a recent study on dementia [38], where healthy older adults (controls) and dementia patients (experimental groups) participated in a laboratory experiment. They had their physiological responses (including the SCL) measured while watching emotionally positive, neutral, or negative video clips for two minutes, and then filled out self-report questionnaires. It was found that healthy older adults’ SCLs decreased by approximately 1.0 μS after watching neutral videos for 120 s; increased by approximately 1.0 μS after watching positive videos for 120 s; remained stable after negative videos for 120 s. They also found that the mean value of their SCLs at the end of the 120 s differed significantly between the two conditions (i.e., 2.0 μS of difference between positive and neutral, 1.0 μS of difference between negative and neutral) [38]. However, had it not been for other physiological or self-report measurements, it would not have been possible to determine whether or not these 1.0–2.0 μS of differences were clinically significant as such, although they were statistically significant (regarding the difference between clinical significance and statistical significance, see [39]). For these reasons, our present study employed self-report questionnaires and interviews and we compared these results with the SCL.

Our LME analysis showed a discrepancy between the surgeons’ physiological and subjective responses; only the experts’ arousal decreased (i.e., their sympathetic nervous system activity reduced), but both the experts and residents reported relaxation after listening to natural sounds. Our results partially contradict the findings of [24]; in that study, more experienced surgeons “did not demonstrate statistically significant increases in SCL” between during rest (before an operation), during open surgery, and during laparoscopic surgery, although “SCL values increased progressively and significantly (*p* < 0.01)” in this order (i.e., rest, open, laparoscopic) for the group as a whole, which suggests that surgery does not affect experienced surgeons’ SCLs. The discrepancy between our results and those of [24] may be due to the different experimental protocols; unlike [24], we had surgeons take a break after an operation. Therefore, in our study, the acute stress derived from the operation may have persisted during the break and affected the surgeons’ SCLs, but the expert surgeons may have been less susceptible to the stress. In that sense, our results could align with those in [24]. The rationales and the details of this interpretation are discussed in the following two paragraphs. 

The absence of residents’ physiological restoration could arise from differences in coping strategies and rumination between the experts and the residents; the former could afford to calm themselves down during the break, whereas the latter may have still been tense. The experts’ answers to Question 1 in the interview (see Table S5 in Supplementary Materials) imply that they were not tense during an operation and did not dwell on their performances afterwards. Distracting thoughts would not have disturbed them during the break. In contrast, the interview results imply that the residents reflected on their performances during the break. For example, as is explained in Surgeon R2′s answer to Q6 in the interview on the day of Trial No. 8 (see Table S7 in Supplementary Materials), the residents sometimes dwelt on their surgical performance even after an operation ended, while neither of the experts mentioned this in the interview. Given that “it is common for SCL to gradually decrease while subjects are at rest” [21], we cannot rule out the possibility that the residents’ reflection impeded their SCL reduction during a break (i.e., activated their sympathetic nervous system) because the residents were sitting without doing anything physically during a break.

Unlike the residents, the experts could clear their minds after a surgical procedure, which enabled the 10-min break (with natural sounds) to reduce their sympathetic nervous system activity. The difference between the experts and the residents could also be explained by the notion of one of the studies conducted by De Bloom, although it targeted at a different population; it suggested that energy management during work and recovery experiences after work are mutually reinforcing [40]. That is, the experts were more used to performing operations and may have been able to manage their energy at work more efficiently than the residents, which may have resulted in the experts’ greater physiological relaxation. To sum up, listening to natural sounds for 10 min after a surgical operation could at least ameliorate both the experts’ and residents’ mood states, although it did not calm the residents physiologically.

This study has some limitations. We could not equalize the magnitude of the surgeons’ stress derived from a surgical operation because no two operations were the same. In addition, as random individual differences or surgeon selection may have played a part in this study with a small number of participants, we cannot generalize the results to all experts or residents. The results may not be generalizable to other types of natural sounds, either. We also had to interrupt or omit occasionally POMS2-A Short or the interview survey when a surgeon was in a hurry. The interview results also reveal a limitation of this study; the preference for natural sounds was not necessarily due to their restorative effect as such, but to other factors, especially when the subject was a resident. The evaluation of natural sounds seemed to depend on a surgeon’s feelings or the surrounding conditions. Surgeon R2 liked to have a break without natural sounds when he wanted to concentrate on thinking about the issues, but he liked to have a break with natural sounds when he tried to take his mind off things. In addition, natural sounds were desirable because of their secondary effects; Surgeon R1, who disliked a break without natural sounds, passively supported natural sounds because the sounds dispelled the discomfort of sitting still without doing anything.

## 5. Conclusions

In this study, our research interest lies in (1) whether listening to natural sounds after a surgical operation reduces surgeons’ physiological and psychological stress and (2) whether surgeons’ stress in this situation proceeds in a different way depending on their level of experience. We aimed to examine all cases in a many-sided way combining self-report and physiological data based on a longitudinal design. We measured surgeons’ SCLs every 5 s continuously during a 10-min break (i.e., 120 measurement occasions) and their self-report data using ROS-J (before an operation, after an operation, and after a break) and POMS2-A Short (after an operation and after a break) and interviewed them (after a break). We also categorized surgeons into two groups according to their levels of experience to observe in-depth how surgeons’ physiological responses and self-report data change over time within each of these groups. This study empirically developed two hypotheses on the physiological and psychological restorative effect of natural sounds on surgeons (representing professionals working in a stressful environment): (H1) Listening to natural sounds immediately after work improves surgeons’ mood states regardless of their levels of experience. (H2) Inexperienced surgeons’ tension persists so long that the restorative effect of a 10-min exposure to natural sounds on their sympathetic arousal was negligible. Future research involving a larger number of surgeons with different levels of expertise could empirically test the hypotheses generated and further develop the findings of this study. The potential beneficiaries of this research will not only be surgeons. This risk-free, easy-to-use way of stress alleviation with natural sounds could benefit highly-stressed people in a wide range of medical professions working indoors.

## Figures and Tables

**Table 1:** Overview of the whole experiment.

Trial No.	Experimental /Control	Date	AM/PM	Surgeon	SCL	ROS-J	POMS2-A Short	Interview
1	Control	8 October 2019	AM	E1_1	x	x	^a^	^b^
2	Control	October 9	AM	E2_1	^c^	x	x	^b^
3	Experimental	October 16	AM	E2_2	x	x	^a^	x
4	Experimental	October 17	AM	E2_3	x	x	x	x
5	Experimental	October 18	AM	E1_2	x	x	x	x
6	Control	November 12	AM	R2_1	^c^	x	x	x
7	Experimental	November 14	AM	R2_2	x	x	x	x
8	Control	November 15	PM	R2_3	x	x	x	x
9	Experimental	November 19	AM	E1_3	x	x	x	x
10	Experimental	November 26	AM	R2_4	x	x	x	x
11	Control	November 27	AM	E2_4	x	x	x	x
12	Experimental	November 28	AM	E2_5	x	x	x	x
13	Experimental	December 4	AM	R2_5	x	x	x	x
14	Experimental	December 5	AM	E2_6	x	x	x	x
15	Control	December 6	AM	R1_1	x	x	x	x
16	Experimental	December 11	AM	R1_2	x	x	x	x
17	Control	December 20	PM	R1_3	x	x	x	x
18	Experimental	14 January 2020	AM	R2_6	x	x	x	x
19	Experimental	January 21	PM	R1_4	x	x	x	x
20	Control	January 22	AM	E1_4	x	x	x	x
21	Experimental	January 28	AM	E1_5	x	x	x	x
22	Experimental	January 31	AM	E1_6	x	x	x	x
23	Experimental	February 5	PM	R1_5	x	x	x	x

Note. AM/PM = an experiment was conducted in the morning or afternoon; x = the measurement was performed. The last one-digit number of each item in the row of “Surgeon” refers to the intraparticipant number of experiments. ^a^ POMS2-A Short was not conducted because the surgeon was in a hurry; ^b^ interview was not conducted because the surgeon was in a hurry; ^c^ SCL was not measured due to the malfunction of the device.

**Table 2:** Parameter estimates for the SCL.

Fixed Effects: Parameter Estimates and 95% CI	
SCL	
	Model 1
$\hat{\beta_{1}}\times NS$	0.69 (−1.55, 2.92)
$\hat{\beta_{2}}\times SC$	−0.46 (−12.78, 11.85)
$\hat{\beta_{3}}\times TIME$	−0.024 ** (−0.026, −0.024)
$\hat{\beta_{4}}\times NS\times TIME$	0.021 ** (0.019, 0.022)
$\hat{\beta_{5}}\times SC\times TIME$	0.022 ** (0.020, 0.024)
$\hat{\beta_{6}}\times NS\times SC\times TIME$	−0.034 ** (−0.036, −0.032)
$\hat{\beta_{0}}\;\left(\mathrm{constant}\right)$	6.46 (2.22, 10.69)
Random effects: Parameter estimates and 95% CI	
$\hat{\sigma_{b_{1i}}}$	2.70 (0.90, 8.12)
$\hat{\sigma_{b_{2ij}}}$	2.15 (1.52, 3.04)
$\hat{\sigma }$	0.44 (0.43, 0.45)
Number of observations	2520

Note. ** *p* < 0.01. *NS*: the experimental condition (with natural sounds); here, the control condition was used as reference. *SC*: expert surgeons; here, resident surgeons were used as reference. The unit of the time variable *TIME* was five seconds.

**Table 3:** Parameter estimates for the ROS-J total score and the POMS2-A Short TMD score.

Fixed Effects: Parameter Estimates and 95% CI					
ROS-J (Total Score)			POMS2-A Short (TMD Score)		
	Model 1	Model 2		Model 1	Model 2
$\hat{\beta_{1}}\times MO1$	1.38 (−2.84, 5.59)	0.08 (−6.37, 6.53)	$\hat{\beta_{1}}\times MO3$	−1.58 (−5.45, 2.29)	−0.56 (−5.77, 4.64)
$\hat{\beta_{2}}\times MO3$	0.25 (−3.96, 4.46)	−0.45 (−6.90, 6.00)	$\hat{\beta_{2}}\times NS$	1.84 (−2.03, 5.71)	1.93 (−3.43, 7.29)
$\hat{\beta_{3}}\times NS$	−1.17 (−4.72, 2.38)	−0.89 (−6.49, 4.70)	$\hat{\beta_{3}}\times SC$		−2.59 (−32.51, 27.32)
$\hat{\beta_{4}}\times SC$		2.46 (−9.28, 14.21)	$\hat{\beta_{4}}\times MO3\times NS$	−5.24 * (−10.15, −0.33)	−7.22 * (−13.99, −0.44)
$\hat{\beta_{5}}\times MO1\times NS$	−0.46 (−5.48, 4.56)	−1.84 (−9.75, 6.07)	$\hat{\beta_{5}}\times MO3\times SC$		−2.47 (−10.40, 5.46)
$\hat{\beta_{6}}\times MO3\times NS$	6.96 ** (1.94, 11.98)	7.86 (−0.049, 15.77)	$\hat{\beta_{6}}\times NS\times SC$		−0.17 (−8.17, 7.83)
$\hat{\beta_{7}}\times MO1\times SC$		−1.13 (−8.87, 6.61)	$\hat{\beta_{7}}\times MO3\times NS\times SC$		4.40 (−5.65, 14.46)
$\hat{\beta_{8}}\times MO3\times SC$		−2.92 (−10.66, 4.82)			
$\hat{\beta_{9}}\times NS\times SC$		−3.17 (−9.72, 3.37)			
$\hat{\beta_{10}}\times MO1\times NS\times SC$		5.21 (−4.04, 14.47)			
$\hat{\beta_{11}}\times MO3\times NS\times SC$		3.70 (−5.56, 12.95)			
$\hat{\beta_{0}}\times Constant$	22.50 ** (19.52, 25.48)	22.43 ** (17.87, 26.99)	$\hat{\beta_{0}}\times Constant$	48.38 ** (42.25, 54.51)	49.66 ** (39.82, 59.50)
Random effects: Parameter estimates and 95% CI					
$\hat{\sigma_{b_{1ij}}}$	0.81 (0.07, 9.31)	0.73 (0.02, 32.98)	$\hat{\sigma_{b_{1ij}}}$	5.09 (2.08, 12.45)	6.07 (2.07, 17.77)
$\hat{\sigma }$	4.05 (3.36, 4.89)	2.14 (1.41, 3.24)	$\hat{\sigma }$	4.35 (3.30, 5.73)	4.52 (3.37, 6.06)
Number of observations	69	69	Number of observations	42	42

Note. * *p* < *0*.05; ** *p* < *0*.01. *NS*: the experimental condition (with natural sounds); here, the control condition was used as reference. *SC*: expert surgeons; here, resident surgeons were used as reference. *MO1*: Measurement Occasion 1 (before an operation). *MO3*: Measurement Occasion 3 (after a break). Here, *MO2* (after an operation) was used as reference.

## Data Availability

Supplementary data and information are available in the Supplementary Materials.

## Supplementary Materials

The following supporting information can be downloaded at: https://www.mdpi.com/article/10.3390/ijerph191912736/s1, Table S1: Parameter estimates for the ROS-J (sub)scales (Model 1); Table S2: Parameter estimates for the ROS-J (sub)scales (Model 2); Table S3: Parameter estimates for the POMS2-A Short (sub)scales (Model 1); Table S4: Parameter estimates for the POMS2-A Short (sub)scales (Model 2); Table S5: Surgeons’ answers to Questions 1–4 in the interview; Table S6: Surgeons’ answers to Question 5 in the interview; Table S7: Surgeons’ answers to Question 6 in the interview; Table S8. Surgeons’ answers to Question 7 and 8 in the interview.
