# Supplementary material for: Alleviating Surgeons’ Stress through Listening to Natural Sounds in a Half-Encapsulated Rest Space after an Operation: A Pilot, Longitudinal Field Study
Source: Int J Environ Res Public Health. 2022 Oct 5;19(19):12736. doi: 10.3390/ijerph191912736 (PMC9564721; doi:10.3390/ijerph191912736)
Supplement: Supplementary file 1 [file ijerph-19-12736-s001.zip › ijerph-1935469-supplementary.pdf]

Supplementary Materials

Article title:

Alleviating surgeons' stress through listening to natural sounds in a half-encapsulated rest space after an operation: A pilot, longitudinal field study

Authors:

Yasushi Suko, Tomoharu Shindo, Kaoru Saito, Norimasa Takayama, Shin'ichi Warisawa, Tetsuya Sakuma, Masaaki Ito, Pasi Kytölä, Tapio Nummi and Kalevi Korpela

Note:

- The section names in this Supplementary Materials correspond to those in the main article.
- All the references mentioned in this file are included in the main article.
- Tables S1-S8 are shown at the end of this Supplementary Materials file.

Table of Contents

Materials and Methods 2

Sample and Experimental Protocol 2

Rationale for the Length of the Break 2

Counterbalancing of the Experiencing Order 2

Results 3

Skin conductance level (SCL) 3

Raw values of the surgeons' SCL 3

Equation 3

ROS-J 4

Equations 4

Sub-scales 5

POMS2-A Short 6

Equations 6

Sub-scales 6

Interview 8

List of Questions 8

Detailed Results 9

Tables S1-S8 11

Materials and Methods

Sample and Experimental Protocol

Rationale for the Length of the Break

The rationale for the length of the break (i.e., 10 minutes) is as follows. Before beginning the experiment, we interviewed several surgeons, including some who did not participate in the experiment, and asked how long breaks they generally took after an operation. Their answers ranged from approximately 10 minutes to 15 minutes, and we chose 10 minutes to make the intervention in our experiment as similar to the surgeons' usual breaks as possible. Having a 15-minute break every time for our experiment would be challenging for surgeons because they had to visit the intensive care unit immediately after having a break to monitor a patient who had undergone their surgical operation.

Counterbalancing of the Experiencing Order

We did not consider counterbalancing the experiencing order of the two conditions (experimental/control) in this study. Although it is needed in a laboratory experiment, where participants experience both conditions immediately one after another, in our experiment, each surgeon took part in a trial only once a day. In addition, it took several days (one day at the shortest, 64 days at the longest) before he participated in the subsequent trial. During such intervals the surgeons were exposed to various stressful situations, such as surgical operations, thus counterbalancing the experiencing order of the two conditions was not considered.

Results

In this section, we explain the details of parameters in the equations for the SCL, ROS-J, and POMS2-A Short. The results for the sub-scales of ROS-J and POMS2-A Short are also described here.

Skin conductance level (SCL)

Raw values of the surgeons' SCL

Figure S1 shows the raw values of the surgeons' SCLs during a 10-minute break in each trial.

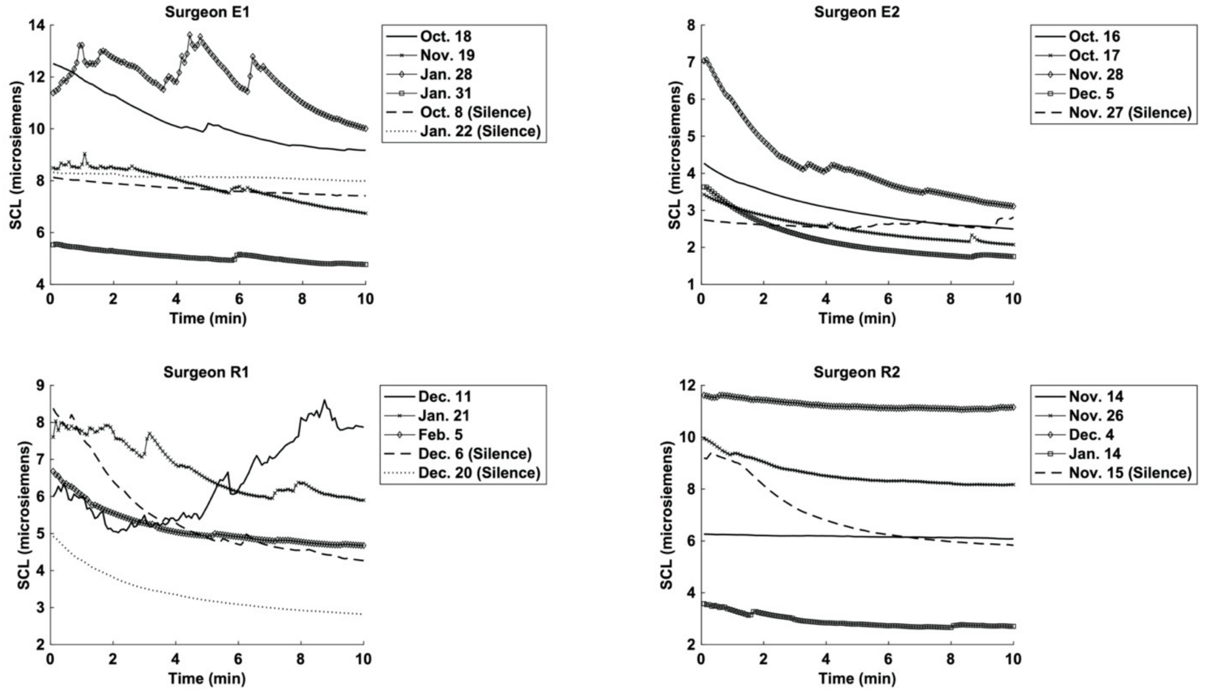

Figure S1. The raw values of the surgeons' SCLs in each trial. Note. Silence = a break without natural sounds.

Equation

In the LME models, the estimations for the SCL are expressed in the following equation:

Equation (1). LME Model for the SCL

$$SCL_{ijk} = \beta_0 + \beta_1 \times NS_i + \beta_2 \times SC_i + \beta_3 \times TIME_i + \beta_4 \times NS_i \times TIME_i + \beta_5 \times SC_i \times TIME_i + \beta_6 \times NS_i \times SC_i \times TIME_i + b_{1i} + b_{2ij} + \epsilon_{ijk}$$

Equation (1) has two random effects; b_{1i} is the grouping at the surgeons' level (expert/resident), and b_{2ij} is the grouping at nested surgeons' working day j level. The interaction terms make it possible to test the hypothesis that the relationship between silence and natural sound on the SCL was different for expert surgeons and resident surgeons.

ROS-J

Equations

In the LME models, the estimations for ROS-J are expressed in the following equations:

Equation (2). LME Model 1 for ROS-J

$$ROSJ_{ijk} = \beta_0 + \beta_1 \times MO1_i + \beta_2 \times MO3_i + \beta_3 \times NS_i + \beta_5 \times MO1_i \times NS_i + \beta_6 \times MO3_i \times NS_i + b_{1ij} + \epsilon_{ijk}$$

Equation (3). LME Model 2 for ROS-J

$$ROSJ_{ijk} = \beta_0 + \beta_1 \times MO1_i + \beta_2 \times MO3_i + \beta_3 \times NS_i + \beta_4 \times SC_i + \beta_5 \times MO1_i \times NS_i + \beta_6 \times MO3_i \times NS_i + \beta_7 \times MO1_i \times SC_i + \beta_8 \times MO3_i \times SC_i + \beta_9 \times NS_i \times SC_i + \beta_{10} \times MO1_i \times NS_i \times SC_i + \beta_{11} \times MO3_i \times NS_i \times SC_i + b_{1ij} + \epsilon_{ijk}$$

In Equations (2) and (3), indices *i*, *j*, and *k* refer respectively to a surgeon, a measurement occasion (*MO*), and repetition in a particular surgeon and *MO*.

Sub-scales

In this study the ROS total score refers to the sum of the scores for its six sub-scales divided by six. Three of the six items reflect relaxation and calmness (Q1: "I feel calm," Q3: "I have enthusiasm and energy for my everyday routines," Q4: "I feel restored and relaxed"), one item reflects attention restoration (Q2: "I can concentrate well and feel alert"), and two items reflect clearing one's thoughts (Q5: "I can forget everyday worries," Q6: "My thoughts are clear") (Korpela et al., 2008).

Table S1 shows the parameter estimates for Model 1. In general, a break with natural sounds significantly increased the surgeons' Q1 scores by 1.28, 95% CI [0.07, 2.49], and Q2 scores by 1.44, 95% CI [0.31, 2.56] (β_6), although it did not change their Q3, Q4, Q5, and Q6 scores significantly.

Table S2 shows the parameter estimates for Model 2. Compared to the residents, the experts' Q3, Q4, Q5, and Q6 scores did not change significantly after a break, regardless of with or without natural sounds (β_8 or β_{11}). The residents' scores for Q1: "I feel calm" significantly increased by 1.70, 95% CI [0.07, 3.33], after a break with natural sounds (β_6), but not after a break without natural sounds (β_2). The experts' scores for Q1 did not change significantly after a break, regardless of with or without natural sounds. The residents' scores for Q2: "I can concentrate well and feel alert" did not change significantly after a break, regardless of with or without natural sounds. The experts' scores for Q2 significantly increased by -1.81 (i.e., decreased by 1.81), 95% CI [-3.37, -0.25], after a break without natural sounds (β_8), but not after

a break with natural sounds (β_{11}). To sum up, in Model 2, the residents' Q1 scores after a break with natural sounds and the experts' Q2 scores after a break without natural sounds changed significantly.

POMS2-A Short

Equations

In the LME models, the estimations for POMS2-A Short are expressed in the following equations:

Equation (4). LME Model 1 for POMS2-A Short

$$POMS2A\ Short_{ijk} = \beta_0 + \beta_1 \times MO3_i + \beta_2 \times NS_i + \beta_4 \times MO3_i \times NS_i + b_{1ij} + \epsilon_{ijk}$$

Equation (5). LME Model 2 for POMS2-A Short

$$\begin{aligned} POMS2A\ Short_{ijk} \\ &= \beta_0 + \beta_1 \times MO3_i + \beta_2 \times NS_i + \beta_3 \times SC_i + \beta_4 \times MO3_i \times NS_i \\ &+ \beta_5 \times MO3_i \times SC_i + \beta_6 \times NS_i \times SC_i + \beta_7 \times MO3_i \times NS_i \times SC_i + b_{1ij} + \epsilon_{ijk} \end{aligned}$$

In Equations (4) and (5), indices i, j, and *k* refer respectively to a surgeon, a measurement occasion (*MO*), and repetition in a particular surgeon and *MO*.

Sub-scales

POMS2-A Short has seven subscales, by which the TMD score is calculated: Anger-Hostility (AH), Confusion-Bewilderment (CB), Depression-Dejection (DD), Fatigue-Inertia (FI),

Tension-Anxiety (TA), Vigor-Activity (VA), and Friendliness (F). Higher values in AH, CB, DD, FI, and TA correspond to negative mood states, whereas those in VA and F correspond to positive mood states. Table S3 shows the parameter estimates for Model 1. In general, a break with natural sounds significantly increased the surgeons' POMS2-A Short CB scores by -4.67 (decreased by 4.67), 95% CI [-8.62, -0.72], FI scores by -5.63, 95% CI [-10.70, -0.57], and VA scores by 7.59, 95% CI [2.13, 13.04] (β_4), although it did not significantly change their AH, DD, TA, and F scores. In contrast, a break without natural sounds did not significantly change any POMS2-A Short scores (β_1).

Table S4 shows the parameter estimates for the Model 2. Neither the experts' nor residents' AH, DD, TA, and F scores changed significantly after a break, regardless of with or without natural sounds.

The residents' CB scores significantly increased by -9.93 (decreased by 9.93) on average, 95% CI [-15.45, -4.42], after a break with natural sounds (β_4), but not after a break without natural sounds (β_1). The experts' CB scores significantly increased by 7.66 on average, 95% CI [0.29, 15.02], after a break with natural sounds (β_7), but not after a break without natural sounds (β_5).

The residents' FI scores significantly decreased by 7.65 on average, 95% CI [-14.12, -1.18], after a break with natural sounds (β_4), but not after a break without natural sounds (β_1). The experts' FI scores did not change significantly after a break, regardless of with or without natural sounds.

The residents' VA scores significantly increased by 8.91 on average, 95% CI [1.63, 16.20], after a break with natural sounds (β_4), but not after a break without natural sounds (β_1).

The experts' VA scores did not change significantly after a break, regardless of with or without natural sounds.

Interview

List of Questions

The main questions that we asked the surgeons during the interview were as follows:

- Q1. Were you nervous during the operation today?
- Q2. Please rate how nervous you were using a 1-10 Likert Scale (1 = not nervous at all to 10 = very nervous)
- Q3. Please rate the difficulty level of today's operation that you expected before it began by a 1-10 Likert Scale (1 = easy to 10 = difficult)
- Q4. Please rate the difficulty level of today's operation that you felt after it finished by a 1-10 Likert Scale (1 = easy to 10 = difficult)
- Q5. Please describe your preference for the type of natural sounds and the volume (this question was asked only in the experimental condition, in which the surgeons listened to natural sounds)
- Q6. Which do you prefer, having natural sounds or not having them during the break?
- Q7. Which do you prefer, resting inside or outside of the Sound Cocoon? Also, would your answer to this question differ depending on whether or not there were natural sounds?
- Q8. How was the duration of the 10-minute break? Was it "long," "moderate," or "short"?

Detailed Results

The surgeons' answers to these eight questions are shown in Tables S5, S6, S7, and S8. As for Q6, "Which do you prefer, having natural sounds or not having them during the break?" Surgeon E1 and R1 preferred "natural sounds" from the beginning of the experiment. Surgeon E1 said, "Listening to natural sounds makes me feel as if I had entered a different world, which changes my mood very much in a positive sense," in his second participation in the experiment (Trial No. 5) and his opinion remained the same throughout the entire experimental period. Surgeon R1 said, "I prefer (a break) 'with natural sounds'" in his first participation (Trial No. 15), and it also remained the same until the end of the entire experimental period. He explained the reason why he thought so on the same day, saying "Were it not for natural sounds, the ambient noise (e.g., the sound of a TV or a vending machine near the Sound Cocoon) would distract me."

In contrast, Surgeon E2 and R2's answers changed as time passed. Surgeon E2 answered, "Whether natural sounds are accompanied or not does not matter for me. Having a break inside of the Sound Cocoon itself would be meaningful because this apparatus insulates sound around here," in his second participation (Trial No. 3). However, he eventually began to prefer a break with natural sounds to a break without natural sounds since his fourth experiment (Trial No. 11). On the next day (Trial No. 12), he explained the reason, saying "Listening to natural sounds makes me feel that my worries are small, but were it not for the sounds, I feel my worries are big." Surgeon R2 said, "I prefer 'without natural sounds.' Today I had a concern, and the break without natural sounds has enabled me to concentrate on thinking about it," in his first participation (Trial No. 6). By contrast, in his third participation (Trial No. 8), Surgeon R2 answered,

> Today I would prefer 'with natural sounds,' but it depends on the day. Natural sounds help me scatter my mind when I am dwelling on my poor performance at operations; were it not for the sounds, I would not be able to get my worries out of my head.

Since that day until the end of the entire experimental period, he continued to say that he preferred to have a break with natural sounds.

Tables S1-S8

Table S1. Parameter estimates for the ROS-J (sub)scales (Model 1).

	Fixed effects: Parameter estimates and 95% CI						
	Total score	Q1	Q2	Q3	Q4	Q5	Q6
$\widehat{\beta_1} \times MO1$	1.38<br>(-2.84, 5.59)	0.00<br>(-1.10, 1.10)	0.13<br>(-0.93, 1.18)	0.88<br>(-0.21, 1.96)	0.50<br>(-0.51, 1.51)	-0.38<br>(-1.51, 0.76)	-0.38<br>(-1.51, 0.76)
$\widehat{\beta_2} \times MO3$	0.25<br>(-3.96, 4.46)	0.50<br>(-0.60, 1.60)	-0.63<br>(-1.68, 0.43)	-0.13<br>(-1.21, 0.96)	0.50<br>(-0.51, 1.51)	0.00<br>(-1.14, 1.14)	0.00<br>(-1.14, 1.14)
$\widehat{\beta_3} \times NS$	-1.17<br>(-4.72, 2.38)	-0.80<br>(-1.66, 0.05)	-0.62<br>(-1.42, 0.17)	0.16<br>(-0.62, 0.94)	0.14<br>(-0.70, 0.98)	0.03<br>(-0.67, 0.73)	0.03<br>(-0.67, 0.73)
$\widehat{\beta_5} \times MO1 \times NS$	-0.47<br>(-5.49, 4.56)	0.25<br>(-0.96, 1.46)	0.19<br>(-0.94, 1.31)	-0.74<br>(-1.84, 0.36)	-0.83<br>(-2.02, 0.35)	0.25<br>(-0.74, 1.25)	0.25<br>(-0.74, 1.25)
$\widehat{\beta_6} \times \mathrm{MO3} \times NS$	6.96**<br>(1.94, 11.98)	1.28*<br>(0.07, 2.49)	1.44*<br>(0.31, 2.56)	1.02<br>(-0.08, 2.12)	1.10<br>(-0.08, 2.29)	0.75<br>(-0.24, 1.74)	0.75<br>(-0.24, 1.74)
$\widehat{\beta_0} \times$ Constant	22.50**<br>(19.52, 25.48)	3.75**<br>(2.97, 4.53)	4.63**<br>(3.88, 5.37)	3.75**<br>(2.99, 4.52)	3.13**<br>(2.41, 3.84)	3.63**<br>(2.82, 4.43)	3.63**<br>(2.82, 4.43)
	Random effects						
	Parameter estimates and 95% CI						
$\widehat{\sigma_{b_{1ij}}}$	0.81<br>(0.07, 9.31)	0.36<br>(0.13, 1.04)	0.38<br>(0.15, 0.97)	0.44<br>(0.20, 0.98)	0.22<br>(0.03, 1.61)	0.57<br>(0.31, 1.06)	0.57<br>(0.31, 1.06)
$\hat{\sigma}$	4.05<br>(3.36, 4.89)	0.98<br>(0.81, 1.18)	0.91<br>(0.75, 1.09)	0.89<br>(0.73, 1.07)	0.96<br>(0.79, 1.15)	0.80<br>(0.66, 0.97)	0.80<br>(0.66, 0.97)
Number of observations	69	69	69	69	69	69	69

Note. *p<.05; **p<.01. Q1: I feel calm, Q2: I can concentrate well and feel alert, Q3: I have enthusiasm and energy for my everyday routines, Q4: I feel restored and relaxed, Q5: I can forget everyday worries, Q6: My thoughts are clear. MO1: Measurement Occasion 1 (before an operation). MO3: Measurement Occasion 3 (after a break). Here, MO2 (after an operation) was used as reference. NS: the experimental condition (with natural sounds); here, the control condition was used as reference.

Table S2. Parameter estimates for the ROS-J (sub)scales (Model 2).

	Fixed effects: Parameter estimates and 95% CI						
	Total score	Q1	Q2	Q3	Q4	Q5	Q6
$\widehat{\beta_1} \times MO1$	0.08	-0.08	0.26	0.75	-0.01	0.08	-0.75
	(-6.37, 6.54)	(-1.72, 1.56)	(-0.84, 1.37)	(-1.17, 2.67)	(-1.34, 1.32)	(-1.43, 1.59)	(-2.05, 0.55)
$\widehat{\beta_2} \times MO3$	-0.45	-0.17	0.26	0.10	-0.23	0.14	-0.29
	(-6.90, 6.01)	(-1.82, 1.47)	(-0.84, 1.36)	(-1.82, 2.02)	(-1.56, 1.10)	(-1.37, 1.65)	(-1.59, 1.01)
$\widehat{\beta_3} \times NS$	-0.89	-0.94	-0.31	0.36	0.45	-0.00	0.12
	(-6.49, 4.70)	(-2.09, 0.21)	(-1.45, 0.82)	(-1.00, 1.72)	(-0.75, 1.65)	(-0.99, 0.98)	(-1.08, 1.32)
$\widehat{\beta_4} \times SC$	2.47	-0.15	1.21	0.20	-0.25	0.58	-0.25
	(-9.28, 14.21)	(-3.54, 3.24)	(-1.58, 4.00)	(-3.50, 3.90)	(-3.23, 2.72)	(-2.62, 3.78)	(-3.27, 2.76)
$\widehat{\beta_5} \times MO1 \times NS$	-1.84	-0.36	-0.13	-1.03	-0.99	-0.50	0.85
	(-9.75, 6.07)	(-1.99, 1.27)	(-1.52, 1.25)	(-2.96, 0.89)	(-2.62, 0.64)	(-1.89, 0.89)	(-0.72, 2.42)
$\widehat{\beta_6} \times MO3 \times NS$	7.86	1.70*	1.15	0.85	1.38	0.57	1.48
	(-0.050, 15.77)	(0.07, 3.33)	(-0.24, 2.53)	(-1.07, 2.78)	(-0.25, 3.00)	(-0.82, 1.96)	(-0.09, 3.05)
$\widehat{\beta_7} \times MO1 \times SC$	-1.13	-0.55	-0.26	0.12	1.00	-0.75	1.99*
	(-8.87, 6.61)	(-2.78, 1.68)	(-1.82, 1.30)	(-2.32, 2.56)	(-0.88, 2.89)	(-2.86, 1.36)	(0.15, 3.83)
$\widehat{\beta_8} \times MO3 \times SC$	-2.92	0.33	-1.81*	-0.57	1.46	-0.30	0.49
	(-10.66, 4.82)	(-1.90, 2.56)	(-3.37, -0.25)	(-3.01, 1.87)	(-0.42, 3.34)	(-2.41, 1.80)	(-1.35, 2.33)
$\widehat{\beta_9} \times NS \times SC$	-3.17	-0.79	-0.65	-0.37	-0.57	0.17	-0.35
	(-9.72, 3.37)	(-2.29, 0.71)	(-2.24, 0.94)	(-1.97, 1.23)	(-2.25, 1.10)	(-1.16, 1.50)	(-2.03, 1.33)
$\widehat{\beta_{10}} \times MO1 \times NS \times SC$	5.21	1.91	0.59	0.69	0.24	1.17	-0.99
	(-4.05, 14.47)	(-0.21, 4.04)	(-1.35, 2.53)	(-1.58, 2.96)	(-2.03, 2.52)	(-0.72, 3.05)	(-3.18, 1.21)
$\widehat{\beta_{11}} \times MO3 \times NS \times SC$	3.70	0.62	0.73	0.42	-0.59	0.43	-0.00
	(-5.56, 12.95)	(-1.51, 2.74)	(-1.20, 2.66)	(-1.85, 2.68)	(-2.86, 1.69)	(-1.45, 2.32)	(-2.20, 2.19)
$\widehat{\beta_0} \times Constant$	22.43**	4.20**	4.01**	3.68**	3.25**	3.29**	3.74**
	(17.87, 26.99)	(3.04, 5.37)	(3.09, 4.93)	(2.32, 5.03)	(2.27, 4.23)	(2.22, 4.36)	(2.75, 4.74)
	Random effects: Parameter estimates and 95% CI						
$\widehat{\sigma_{b_{1ij}}}$	0.73	0.48	0.06	0.57	0.15	0.51	0.18
	(0.02, 32.98)	(0.18, 1.27)	(0.00, 3.96e+3)	(0.26, 1.23)	(0.00, 21.25)	(0.23, 1.14)	(0.01, 4.96)
$\hat{\sigma}$	2.14	0.64	0.92	0.63	0.95	0.63	0.96
	(1.41, 3.24)	(0.39, 1.05)	(0.75, 1.12)	(0.42, 0.94)	(0.79, 1.16)	(0.43, 0.93)	(0.79, 1.16)
Number of observations	69	69	69	69	69	69	69

Note (Table S2). *p<.05; **p<.01. Q1: I feel calm, Q2: I can concentrate well and feel alert, Q3: I have enthusiasm and energy for my everyday routines, Q4: I feel restored and relaxed, Q5: I can forget everyday worries, Q6: My thoughts are clear. MO1: Measurement Occasion 1 (before an operation). MO3: Measurement Occasion 3 (after a break). Here, MO2 (after an operation) was used as reference. NS: the experimental condition (with natural sounds); here, the control condition was used as reference. SC: expert surgeons; here, resident surgeons were used as reference.

Table S3. Parameter estimates for the POMS2-A Short (sub)scales (Model 1).

	Fixed effects: Parameter estimates and 95% CI							
	AH	CB	DD	FI	TA	VA	F	TMD
$\widehat{\beta_1} \times MO3$	1.67<br>(-0.50, 3.84)	1.14<br>(-2.13, 4.41)	-0.24<br>(-1.99, 1.52)	-3.27<br>(-8.49, 1.95)	-4.20<br>(-9.88, 1.48)	-2.88<br>(-8.03, 2.28)	-0.69<br>(-10.25, 8.88)	-1.58<br>(-5.45, 2.29)
$\widehat{\beta_2} \times NS$	0.49<br>(-1.46, 2.44)	2.88*<br>(0.05, 5.70)	0.93<br>(-0.67, 2.52)	1.93<br>(-2.42, 6.28)	3.38<br>(-1.32, 8.09)	-0.26<br>(-4.77, 4.25)	1.83<br>(-3.40, 7.06)	1.84<br>(-2.03, 5.71)
$\widehat{\beta_4} \times MO3 \times NS$	-2.63<br>(-5.38, 0.13)	-4.67*<br>(-8.62, -0.72)	-1.01<br>(-3.26, 1.24)	-5.63*<br>(-10.70, -0.57)	-3.96<br>(-10.56, 2.63)	7.59*<br>(2.13, 13.04)	-0.06<br>(-7.45, 7.34)	-5.24*<br>(-10.15, -0.33)
$\widehat{\beta_0} \times Constant$	41.51**<br>(37.12, 45.91)	45.48**<br>(38.95, 52.02)	47.11**<br>(38.21, 56.01)	52.05**<br>(46.43, 57.66)	48.48**<br>(42.17, 54.79)	46.95**<br>(42.19, 51.71)	45.39**<br>(38.63, 52.15)	48.38**<br>(42.25, 54.51)
	Random effects: Parameter estimates and 95% CI							
$\widehat{\sigma_{b_{1ij}}}$	3.96<br>(1.67, 9.40)	5.97<br>(2.53, 14.09)	8.59<br>(3.70, 19.95)	2.39<br>(0.69, 8.35)	4.73<br>(1.84, 12.16)	2.04<br>(0.69, 6.06)	5.00<br>(2.47, 10.11)	5.09<br>(2.09, 12.45)
$\hat{\sigma}$	4.00<br>(2.46, 6.53)	2.24<br>(1.36, 3.68)	3.83<br>(2.41, 6.08)	6.80<br>(4.31, 10.74)	2.77<br>(1.60, 4.81)	5.79<br>(4.31, 7.78)	4.42<br>(2.65, 7.39)	4.35<br>(3.30, 5.73)
Number of observations	42	42	42	42	42	42	42	42

Note. *p<.05; **p<.01. AH: Anger-Hostility, CB: Confusion-Bewilderment, DD: Depression-Dejection, FI: Fatigue-Inertia, TA: Tension-Anxiety, VA: Vigor-Activity, F: Friendliness, TMD: Total Mood Disturbance. MO3: Measurement Occasion 3 (after a break). Here, MO2 (after an operation) was used as reference. NS: the experimental condition (with natural sounds); here, the control condition was used as reference. SC: expert surgeons; here, resident surgeons were used as reference.

Table S4. Parameter estimates for the POMS2-A Short (sub)scales (Model 2).
Note. *p<.05; **p<.01. AH: Anger-Hostility, CB: Confusion-Bewilderment, DD: Depression-Dejection, FI: Fatigue-Inertia, TA: Tension-Anxiety, VA: Vigor-Activity,

	Fixed effects: Parameter estimates and 95% CI							
	AH	CB	DD	FI	TA	VA	F	TMD
$\widehat{\beta_1} \times MO3$	1.89<br>(-0.28, 4.06)	3.12<br>(-1.17, 7.42)	0.00<br>(-2.02, 2.02)	-3.93<br>(-10.97, 3.11)	-4.87<br>(-14.12, 4.38)	-6.07<br>(-12.28, -0.15)	0.20<br>(-14.48, 14.88)	-0.56<br>(-5.77, 4.64)
$\widehat{\beta_2} \times NS$	-0.07<br>(-2.03, 1.91)	6.94**<br>(3.03, 10.84)	0.97<br>(-0.87, 2.81)	0.69<br>(-5.02, 6.41)	6.98<br>(-1.27, 15.23)	1.24<br>(-4.88, 7.37)	4.00<br>(-2.48, 10.49)	1.93<br>(-3.43, 7.29)
$\widehat{\beta_3} \times SC$	2.81<br>(-16.79, 22.40)	-0.63<br>(-32.34, 31.08)	-5.95<br>(-48.32, 36.42)	-5.67<br>(-33.76, 22.42)	-2.68<br>(-32.85, 27.49)	-1.01<br>(-20.74, 18.72)	1.21<br>(-32.07, 34.48)	-2.59<br>(-32.51, 27.32)
$\widehat{\beta_4} \times MO3 \times NS$	-2.10<br>(-4.88, 0.68)	-9.93**<br>(-15.45, -4.42)	-1.08<br>(-3.68, 1.52)	-7.65*<br>(-14.12, -1.18)	-9.19<br>(-20.84, 2.47)	8.91*<br>(1.63, 16.20)	-4.01<br>(-13.17, 5.16)	-7.22*<br>(-13.99, -0.44)
$\widehat{\beta_5} \times MO3 \times SC$	-2.03<br>(-8.85, 4.78)	-3.12<br>(-9.09, 2.84)	-1.27<br>(-6.17, 3.62)	0.78<br>(-9.56, 11.13)	1.59<br>(-10.11, 13.28)	7.36<br>(-1.98, 16.70)	-3.48<br>(-25.76, 18.80)	-2.47<br>(-10.40, 5.46)
$\widehat{\beta_6} \times NS \times SC$	4.65<br>(-1.11, 10.41)	-5.93*<br>(-11.16, -0.69)	-0.33<br>(-4.67, 4.02)	2.34<br>(-6.09, 10.76)	-3.64<br>(-13.71, 6.43)	-2.97<br>(-12.03, 6.11)	-5.96<br>(-16.92, 5.01)	-0.17<br>(-8.17, 7.83)
$\widehat{\beta_7} \times MO3 \times NS \times SC$	-3.36<br>(-11.43, 4.71)	7.66*<br>(0.29, 15.02)	0.53<br>(-5.57, 6.64)	4.57<br>(-5.08, 14.23)	5.53<br>(-8.68, 19.73)	-3.69<br>(-14.54, 7.16)	10.16<br>(-5.35, 25.67)	4.40<br>(-5.65, 14.46)
$\widehat{\beta_0} \times Constant$	39.75**<br>(33.92, 45.59)	45.43**<br>(34.72, 56.14)	50.37**<br>(36.25, 64.48)	54.80**<br>(45.70, 63.89)	49.31**<br>(38.84, 59.77)	47.49**<br>(41.26, 53.73)	45.33**<br>(34.95, 55.71)	49.66**<br>(39.82, 59.50)
	Random effects: Parameter estimates and 95% CI							
$\widehat{\sigma_{b_{1ij}}}$	3.87<br>(1.33, 11.29)	7.01<br>(2.53, 19.44)	9.62<br>(3.51, 26.36)	2.47<br>(0.65, 9.36)	5.66<br>(1.88, 17.01)	1.37<br>(0.18, 10.21)	6.19<br>(2.76, 13.84)	6.07<br>(2.07, 17.77)
$\hat{\sigma}$	3.29<br>(1.99, 5.43)	2.21<br>(1.36, 3.59)	3.99<br>(2.50, 6.38)	7.91<br>(4.18, 14.95)	2.77<br>(1.57, 4.89)	5.98<br>(4.32, 8.27)	4.50<br>(2.49, 8.14)	4.52<br>(3.37, 6.06)
Number of observations	42	42	42	42	42	42	42	42

F: Friendliness, TMD: Total Mood Disturbance. MO3: Measurement Occasion 3 (after a break). Here, MO2 (after an operation) was used as reference. NS: the

experimental condition (with natural sounds); here, the control condition was used as reference. SC: expert surgeons; here, resident surgeons were used as reference.

Table S5. Surgeons' answers to Questions 1-4 in the interview.

Surgeon	Trial No.	Q1. Were you nervous during the operation today?	Q2. Degree of nervousness (1 = *easy*, 10 = *difficult*)	Q3. Degree of the expected difficulty of the operation (1 = *easy*, 10 = *difficult*)	Q4. Degree of the actual difficulty of the operation (1 = *easy*, 10 = *difficult*)
E1_1	1	-	-	-	-
E1_2	5	-	-	-	-
E1_3	9	No.	3	7	7
E1_4	20	No.	4	6	6
E1_5	21	Not so much.	4	6	7
E1_6	22	I was nervous at first but calmed down from the middle.	4	6	4
E2_1	2	-	-	-	-
E2_2	3	-	-	-	-
E2_3	4	-	-	-	-
E2_4	11	Not so much.	8	8	8
E2_5	12	Not so much.	6	9	9
E2_6	14	Not so much.	6	7	6
R1_1	15	Not so much.	4	3	3
R1_2	16	Not so much.	3	3	3
R1_3	17	Not so much.	4	4	4
R1_4	19	Yes.	7	3	4
R1_5	23	Yes, a little.	3	4	4
R2_1	6	Yes, fairly.	7	-	-
R2_2	7	Yes, especially during the latter half of the operation.	9	-	-
R2_3	8	Yes.	7	-	-
R2_4	10	Yes.	7	5	6
R2_5	13	Yes, fairly.	6	5	6
R2_6	18	Yes, I was very nervous.	9	5	7

Table S6. Surgeons' answers to Question 5 in the interview

Surgeon	Trial No.	Q5. Please describe your preference for the type of natural sounds and the volume (This question was asked only in the experimental condition).
E1_1	1	-
E1_2	5	I liked birdsong today. The default was the sound of river flowing, but it was blurry, so I changed it to birdsong, which was clear.
E1_3	9	I liked birdsong today. The default was the sound of the wind, but it was not relaxing, so I changed it to the sound of the river flowing. However, it was a bit noisy, so I decided to listen to birdsong. The birdsong was pleasant.
E1_4	20	-
E1_5	21	I preferred the sound of rain today because something sad happened besides the operation. I have chosen the sound of rain to get rid of unpleasant feelings from my mind.
E1_6	22	Today, the ambient sound was a bit noisy, so I listened to natural sounds at a loud volume to mask the surrounding noise. I have chosen the sound of wind because it was continuous. Birdsongs were uneven (sometimes eventful, but sometimes calm), which I did not prefer much today. I did not choose the sound of rain either because it would make me feel gloomy, although it was also continuous.
E2_1	2	-
E2_2	3	I did not change the volume.
E2_3	4	-
E2_4	11	-
E2_5	12	A louder volume might be better. The sound of rain goes in one ear and comes out the other even if I listen to it at a loud volume. I would prefer a stable sound.
E2_6	14	I have chosen the sound of the river flowing because it was noisier in the room than usual. I do not pay much attention to the stationariness of natural sounds.
R1_1	15	-
R1_2	16	I liked "Wind" the most because it was stationary. I feel that stationary sound would be more relaxing than those that were not. I did not change the volume. "River" was noisy.
R1_3	17	-
R1_4	19	I tried to listen to birdsongs after a long time, but I stopped doing so because they were noisy. I have chosen "Wind," which was stationary.
R1_5	23	"Rain" was most relaxing. "Wind" was also good. I have not listened to "Bird 1" and "Bird 2" recently because they are noisy. I did not change the volume today. I did not listen to "River." I no longer remember what kind of sound "River" is.
R2_1	6	-
R2_2	7	The default volume was moderate. The natural sounds were also pleasant.
R2_3	8	-
R2_4	10	I have listened to "Rain" and "River." I did not like the popping sound of "Rain," so I changed it to "River" and continued to listen to it until the end of the listening period. I decreased the volume a bit. I listened to these two sounds because they were at the top of the playlist. I would have chosen other sounds if the playlist was shuffled. I personally like the sound of water. If there were 20 types of sounds available, I would have across scrolled the list and chosen one. But this time I chose from the top of the list because there were only five sounds.
R2_5	13	After having listened to all the sounds, I concluded that "River" in a low volume would be most relaxing.
R2_6	18	I liked "Bird 2" the most today. I decreased the volume a bit from the default because it was a bit noisy.

Table S7. Surgeons' answers to Question 6 in the interview.

Surgeon	Trial No.	Q6. Which do you prefer, with or without natural sounds during the break?
E1_1	1	-
E1_2	5	Listening to natural sounds make me feel as if I had entered a different world, which changes my mood in a positive sense. By contrast, I do not feel as if I had entered a different world when natural sounds are not accompanied.
E1_3	9	I prefer "with natural sounds."
E1_4	20	I prefer "with natural sounds." Time passes slowly without natural sounds. This condition makes me feel as if I were in custody, which makes me tired. Also, I want to get out of the Sound Cocoon when natural sounds are not accompanied because it is boring just to sit still.
E1_5	21	I prefer "with natural sounds."
E1_6	22	I prefer "with natural sounds."
E2_1	2	-
E2_2	3	Whether natural sounds are accompanied or not does not matter for me. Resting inside of the Sound Cocoon itself would be meaningful because this apparatus insulates sound around here. If I am forced to say, I would prefer "with natural sounds" because these sounds mask ambient noise (e.g., other persons' conversations or the sound of a TV in the resting room).
E2_3	4	I would prefer "with natural sounds" in the sense that these sounds mask the ambient noise. Today's break in the experimental condition was not so different from the previous ones.
E2_4	11	I prefer "with natural sounds" because I cannot help but thinking unnecessary things when resting without listening to natural sounds.
E2_5	12	I prefer "with natural sounds." Listening to natural sounds makes me feel that my worries are small, but were it not for the sounds, I feel my worries are big.
E2_6	14	I prefer "with natural sounds."
R1_1	15	I prefer "with natural sounds." Were it not for natural sounds, the ambient noise (e.g., the sound of a TV or a vending machine near the Sound Cocoon) would distract me.
R1_2	16	I prefer "with natural sounds."
R1_3	17	I prefer "with natural sounds." Today's break without natural sounds was not comfortable.
R1_4	19	I prefer "with natural sounds."
R1_5	23	I prefer "with natural sounds."
R2_1	6	I prefer "without natural sounds." Today I had a concern, and the break without natural sounds has enabled me to concentrate on thinking about it.
R2_2	7	It depends on situations. Last time, I preferred "without natural sounds" because no one was around here. However, now I would prefer "with natural sounds" because there are many people around here.
R2_3	8	Well, let's see. Today I would prefer "with natural sounds," but it depends on the day. Natural sounds help me scatter my mind when I am dwelling on my poor performance at operations; were it not for the sounds, I would not be able to get my worries out of my head.
R2_4	10	I prefer "with natural sounds."
R2_5	13	I prefer "with natural sounds."
R2_6	18	I prefer "with natural sounds."

Table S8. Surgeons' answers to Question 7 and 8 in the interview.

Surgeon	Trial No.	Q7. Which do you prefer, resting inside or outside of the Sound Cocoon? Also, would your answer to this question differ depending on with or without natural sounds?	Q8. How was the duration of the 10-minute break? Was it "long," "moderate," or "short"?
E1_1	1	-	-
E1_2	5	-	-
E1_3	9	I prefer inside.	Long.
E1_4	20	I prefer inside if I can listen to natural sounds, but I prefer outside if the sounds are not accompanied.	Long.
E1_5	21	I prefer inside.	A bit short. Today I wish I could have rested a bit longer because I had something sad happen and I wanted to get rid of unpleasant feelings from my mind.
E1_6	22	I prefer inside.	-
E2_1	2	-	-
E2_2	3	-	-
E2_3	4	-	-
E2_4	11	I prefer inside.	Moderate.
E2_5	12	I prefer inside.	Moderate.
E2_6	14	I prefer inside even if natural sounds are not accompanied.	Moderate.
R1_1	15	I prefer inside even if natural sounds are not accompanied.	Long. I felt so because there was no natural sound. I could not help but looking at my watch several times during the break.
R1_2	16	I prefer inside.	Moderate.
R1_3	17	I prefer inside even if natural sounds are not accompanied.	Long.
R1_4	19	I prefer inside.	Might have been moderate. I would have felt like "Hasn't the break finished yet?" during a long break, but today the break finished just when I thought that it would be over soon.
R1_5	23	I prefer inside if I can listen to natural sounds. If the sounds are not accompanied, I would prefer taking a nap in a napping room, rather than just sitting still in a resting room.	Short.
R2_1	6	I prefer inside because the Sound Cocoon insulates external noise.	-
R2_2	7	I prefer inside; it's more relaxing. If I am resting outside of the Sound Cocoon, I may find something that should be done against my will.	A bit long.
R2_3	8	I can't say either. On the day when I couldn't do well during an operation, I may feel restless if I am told to sit still in a small place like the Sound Cocoon.	Moderate.
R2_4	10	I prefer inside even if natural sounds are not accompanied.	A bit long. I almost fell asleep during the break. A seven to eight-minute break would be moderate.
R2_5	13	I prefer inside even if natural sounds are not accompanied, but I would go to a napping room when I want to lie down.	Moderate.
R2_6	18	I prefer inside. If I can't rest inside the Sound Cocoon, I would take a nap in a napping room.	Moderate. I would fall asleep if the break is longer than it is now.
